# Introductory Dental Profession of the West and South

**Published:** 1856

**Authors:** 

**Affiliations:** No. 38 West Fourth street, Cincinnati, Ohio; No. 38 West Fourth street, Cincinnati, Ohio


### INTRODUCTORY

TO THE

### DENTAL PROFESSION

OF THE

### WEST AND SOUTH.

"Once more upon the water! Yet once more!
And the waves bound beneath me as a steed
That knows his rider. Welcome to their roar."

In this, our first appearance in the proprietorship of a Dental Depot, our natural modesty would dictate that we should cast ourselves upon your indulgence and generosity, and this, we doubt not, would be freely accorded. We do not, however, enter the arena of trade without due deliberation and advisement. We have been met with warnings and persuasions, from different quarters, picturing, on the one hand, the competition to be encountered and the difficulties to be surmounted, and, on the other hand, showing us the want of a Depot at several distant points where there is no competition, and where a "golden harvest" would be the sure result of our efforts. To those kind friends we return our sincere thanks, but must beg to be excused from following their suggestions.

We know (and so does every one at all familiar with the trade) that not more than *one-third* of the business in dental materials, properly belonging to Cincinnati, is transacted here. Why this is so, we will let others answer. We think we can offer such inducements as will attract at least *what belongs here*.

For hundreds of miles, to the east, west, north and south, Cincinnati is naturally, and by its numerous railroad and river connections and communications, the *great central commercial point* from whence should be supplied all the mercantile commodities needed by the community, and there is no good reason why these natural advantages should not be made available to the Dental profession, by at once removing every occasion for visiting the East. This can be done by constantly keeping a large and well selected stock of everything used by the Dentist, and by keeping fully posted up in the *progress and improvements* of the profession, promptly supplying every new instrument, apparatus or material, as soon as its practicability is established. This it is our intention to do, and we have a *generous confidence* that we will be sustained by a Liberal Profession.

If a Dentist, anywhere west of the Alleghany Mountains, wishes to visit a city for supplies, he can come here and return quicker and at less expense than he could go to any Eastern city. He can receive his goods sooner and at less expense, and can buy equally cheap, and some kinds cheaper. Then why waste both *time and money.*

"Competition is the life of trade," and when fairly and honorably conducted, you may "consider us in;" but when it becomes necessary to pander to sectarian prejudice, political passions, or sectional feelings, "consider us out"!!

Our object in this enterprise, is to make money! This, we believe, can be done most effectually by taking the lead of everything pertaining to the Instruments, apparatus and materials needed by the profession; keeping the largest stock and greatest variety of goods; furnishing the *best articles* for the *least money*; being at all times ready to supply everything kept by any other establishment, besides some things not to be found elsewhere.

It becomes the interest of every dentist in the South and West to deal with us:

If we can supply Instruments, Materials and Aparatus of improved quality, style and utility, at as low or lower prices than they can be obtained elsewhere;

If we can supply any and everything the Dentist wants;

If all delays and disappointments can be avoided;

If we can furnish goods with *less delay* and at *less expense* of freight, etc.

All these, we are confident, can be done, and therefore it needs no argument to show where your *interests* are.

We expect to *make money* by making it the interest of every dentist in the South and West to deal with us, as it must be apparent to every reflecting mind that we can make more money by selling $50,000 worth of goods at ten per cent. than we could to sell only $25,000 worth at fifteen per cent.

In this issue we can not give a full list of the articles kept, because much of the space which should be devoted to that is occupied by a report of the proceedings of "Mississippi Valley Association of Dental Surgeons," but bear in mind that we have a *full stock* of *everything* needed by the profession, and hold ourselves in readiness to manufacture or procure, either *here or elsewhere*, anything new or not in our line of business, at short notice and reasonable terms.

With kind greetings, hoping to hear from you often, and have the pleasure of seeing you, soon, we are, very respectfully your obedient servants,

TOLAND & THORNE,

No. 38 West Fourth street, Cincinnati, Ohio.

CINCINNATI, March 1, 1856.

